# Cellular Behavior of Human Adipose-Derived Stem Cells on Wettable Gradient Polyethylene Surfaces

**DOI:** 10.3390/ijms15022075

**Published:** 2014-01-28

**Authors:** Hyun Hee Ahn, Il Woo Lee, Hai Bang Lee, Moon Suk Kim

**Affiliations:** 1 Department of Neurosurgery, Catholic University of Korea, Daejeon 301-723, Korea; E-Mails: windy818@hanmail.net (H.H.A.); leeilwoo@catholic.ac.kr (I.W.L.); 2 Department of Molecular Science and Technology, Ajou University, Suwon 443-759, Korea; E-Mail: hblee@ajou.ac.kr

**Keywords:** surface wettability, roughness, corona discharge, human adipose derived stem cells, integrin beta 1 gene, heat shock protein

## Abstract

Appropriate surface wettability and roughness of biomaterials is an important factor in cell attachment and proliferation. In this study, we investigated the correlation between surface wettability and roughness, and biological response in human adipose-derived stem cells (hADSCs). We prepared wettable and rough gradient polyethylene (PE) surfaces by increasing the power of a radio frequency corona discharge apparatus with knife-type electrodes over a moving sample bed. The PE changed gradually from hydrophobic and smooth surfaces to hydrophilic (water contact angle, 90º to ~50º) and rough (80 to ~120 nm) surfaces as the power increased. We found that hADSCs adhered better to highly hydrophilic and rough surfaces and showed broadly stretched morphology compared with that on hydrophobic and smooth surfaces. The proliferation of hADSCs on hydrophilic and rough surfaces was also higher than that on hydrophobic and smooth surfaces. Furthermore, integrin beta 1 gene expression, an indicator of attachment, and heat shock protein 70 gene expression were high on hydrophobic and smooth surfaces. These results indicate that the cellular behavior of hADSCs on gradient surface depends on surface properties, wettability and roughness.

## 1. Introduction

The goal of tissue engineering is to repair damaged tissues and replace injured body parts [1–3]. Scaffolding plays the most central role in these treatments because most of the transplanted cells are anchor dependent and most biological reactions take place on surfaces [4–9]. A basic understanding of the interactions between cells and scaffold surfaces is important for the design of implantable scaffolds. Thus, studies examining these interactions must be conducted. Determining how to modify scaffold surfaces in the construction of implantable scaffolds is also important. Many surface modification techniques are available, including chemical modification with engrafted functional groups, physical modification with low-temperature plasma ion injection, and corona discharge [10,11].

We developed a method for preparing uniformly wetted surfaces through plasma exposure and have reported the cellular behavior and binding of biologic molecules on the polymer surface [11–13]. These surface modifications required repeat experiments for a full evaluation of biologic activities on the surface of uniformly modified biomaterials.

**Figure 1.**
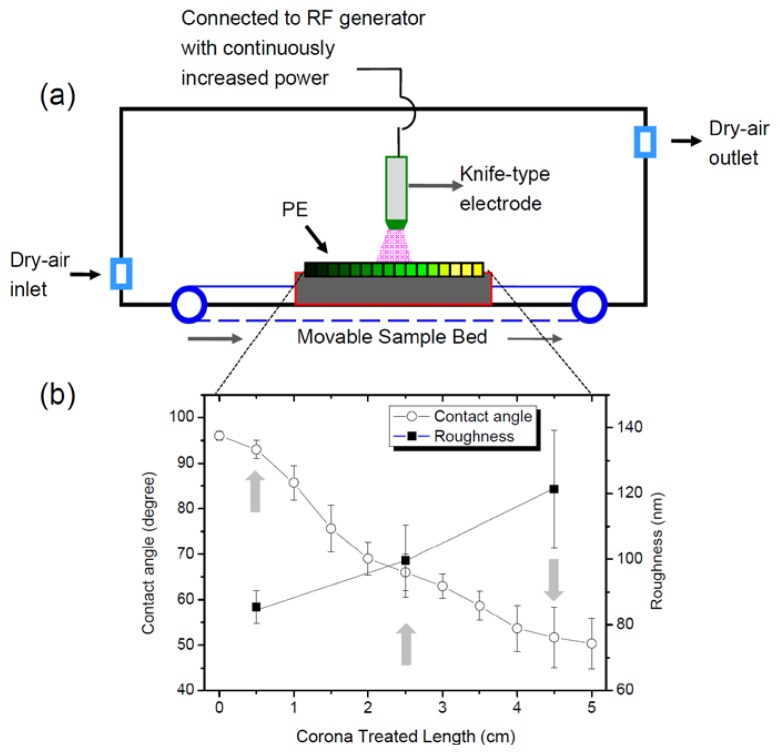
(**a**) Schematic diagram of the corona discharge apparatus used to prepare gradient surfaces; and (**b**) water contact angle *versus* corona-treated polyethylene (PE) length. RF, radio frequency.

Thus, we and other researchers have focused on developing wettability gradient surfaces [10,14–18]. A gradient surface is a surface on which a continuously varying chemical or physical property exists along its length. If biologic molecules are introduced to a gradient surface, they display various behaviors throughout a single experiment. Thus, gradient surfaces have the advantage of reducing the number of experimental errors that might occur with separate experiments [10,14–18]. Our group used a homemade corona discharge to design wettability and rough gradient surfaces as shown in Figure 1a [14–21]. The power of the radio frequency corona discharge apparatus with knife-type electrodes was increased gradually using an automatic motorization drive. This apparatus can easily be used under atmospheric conditions and provides a gradient surface for oxygen-containing functional groups.

Adult stem cells are self-renewing and have the capacity to differentiate into many cell types [22], and research conducted using these cells avoids the ethical controversy surrounding the use of embryonic stem cells. Thus, the use of adult stem cells for cell therapy has been widely investigated. Adipose-derived stem cells (ADSCs) are a type of adult mesenchymal stem cell with the potential for multi-lineage differentiation to chondrocytes, myocytes, adipocytes, or osteoblasts [23–25]. ADSCs are abundant and easy to obtain and can be cultured without supplementary cytokines. ADSCs also display properties similar to those of bone marrow-derived stem cells (BMSCs), which are the most broadly identified adult stem cells. Furthermore, ADSCs comprise a more homogeneous cell population and are easier to maintain than BMSCs. Therefore, ADSCs are considered an ideal source of adult stem cells for tissue engineering and regenerative medicine.

Accordingly, we investigated the cellular behavior of human ADSCs (hADSCs) on wettable and rough gradient surfaces and examined the environment for cell growth and proliferation through evaluation of gene expression of integrin beta 1 (IGb) and heat shock protein 70 (HSP70), which are related to cell attachment and inducement of stress and apoptosis, respectively [26–29]. The results of these investigations will expand understanding of the cellular behavior of ADSCs on wettable and rough surfaces and inform the construction of an implantable scaffold.

## 2. Results

### 2.1. Characterization of Gradient PE Surfaces

**Figure 2.**
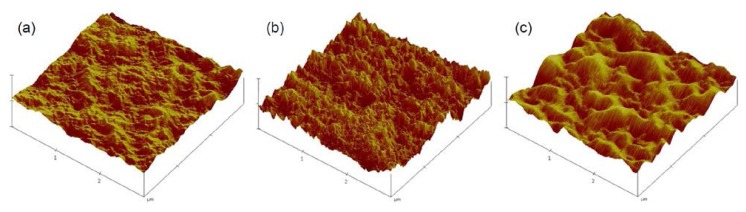
Atomic force microscopy (AFM) images of (**a**) L-0.5 (93º); (**b**) L-2.5 (65º) and (**c**) L-4.5 (52º).

To investigate the cellular behavior of ADSCs on gradient surfaces, a PE sheet was exposed to an RF corona discharge apparatus with increasing power, which provided various oxygen-based polar functional groups such as hydroxyl group, ether, ketone, aldehyde, carboxylic acid, and carboxylic ester, and gradient concentration change of functional group on PE surface [17–21]. Figure 1b shows that the water contact angle of the PE surface changed gradually from 95º to 50º. For further analysis, we examined the topographic image of each PE surface after corona discharge treatment. AFM showed that the corona discharge treatment changed the roughness of the PE surface; specifically, the PE surface topography was changed from 85 to 120 nm by corona discharge treatment (Figures 1b and 2). These results indicate that the PE surface gained more hydrophilic and rough properties after gradual exposure to the corona discharge treatment.

Thus, to characterize cellular behavior of ADSCs, we examined the contact angle of the PE surface at 0.5 (L-0.5), 2.5 (L-2.5), and 4.5 (L-4.5) cm after corona treatment, with water contact angles of approximately 93º (hydrophobic surface), 65º (moderate surface), and 52º (hydrophilic surface), respectively.

### 2.2. Cell Adhesion and Proliferation on Gradient PE Surfaces

**Figure 3.**
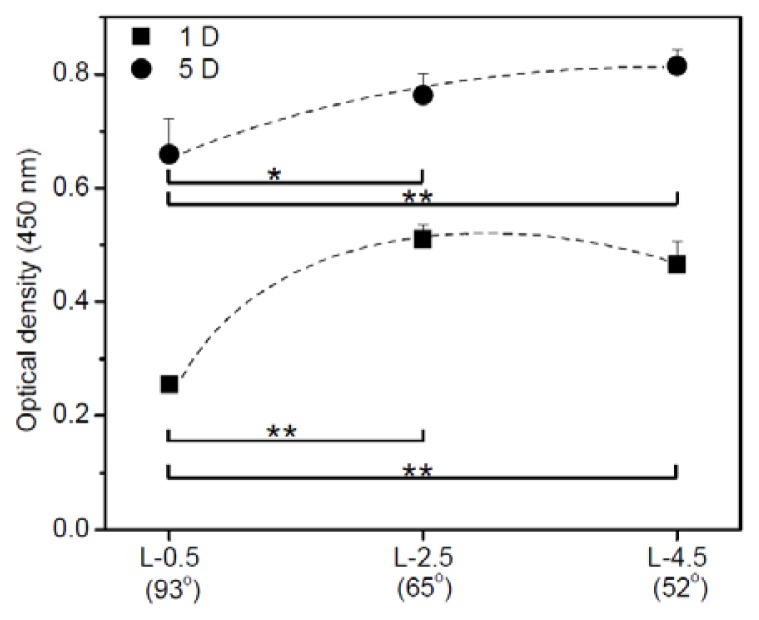
Human adipose-derived stem cell (hADSC) viability measured with a cell counting kit assay. hADSCs grown on each gradient surface (at 0.5 [L-0.5], 2.5 [L-2.5], and 4.5 [L-4.5] cm) were measured on days 1 and 5. Statistical analysis was performed using 1-way analysis of variance with Bonferroni’s multiple comparison (^*^ *p* < 0.01; ^**^ *p* < 0.001).

Figure 3 shows the cell proliferation on gradient surfaces. On day 1, hADSCs attached in greater numbers at L-2.5 than at L-0.5 and in slightly greater numbers than that at L-4.5 (hydrophobic and hydrophilic surfaces, respectively). The plot of attachment at 1 D *versus* water contact angle was parabolic. hADSCs at L-2.5 and L-4.5 showed nearly identical proliferation in culturing between days 1 and 5. The highest number of cells adhered when the water contact angle was below 65º. Lower cell adhesion occurred on surfaces with contact angles of 90º.

**Figure 4.**
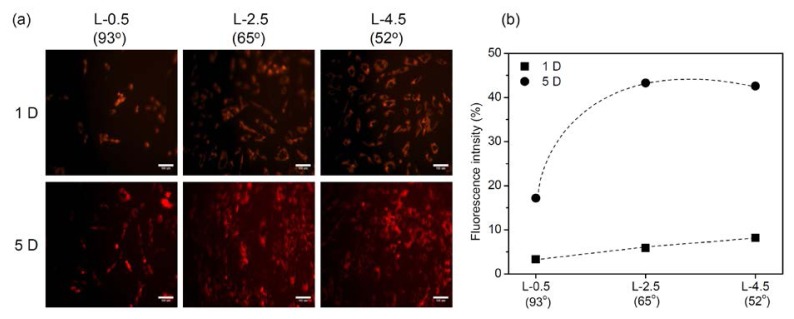
(**a**) Fluorescence images and (**b**) fluorescence intensities of PKH red-labeled hADSCs on each gradient surface (L-0.5, L-2.5, and L-4.5) on days 1 and 5 (magnification: 100×; scale bar: 100 μm).

Figure 4 shows PKH red-labeled hADSCs and their fluorescence intensities on gradient PE surfaces. The red fluorescence expression indicated that the hADSCs attached and proliferated on gradient PE surfaces. The fluorescence intensity showed that the expression on the gradient surfaces at L-2.5 and L-4.5 was higher than that at L-0.5 and increased as culturing time increased. The number of hADSCs adhering to the gradient PE surface apparently increased gradually as the water contact angle decreased and the roughness increased.

**Figure 5.**
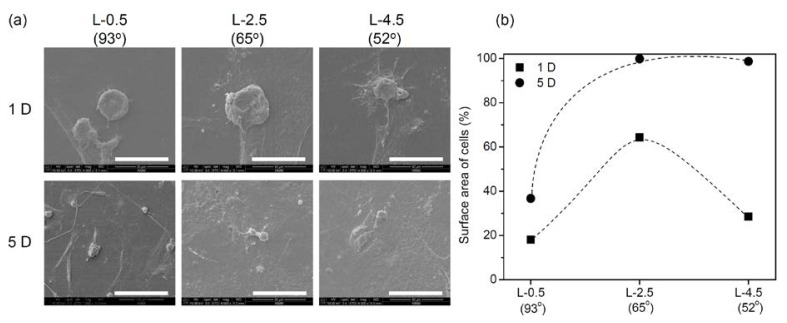
(**a**) Scanning electron microphotographs and (**b**) surface areas of hADSCs adhered to each gradient surface (L-0.5, L-2.5, and L-4.5) on days 1 and 5 (magnification: 4000×; scale bar: 30 μm).

Figure 5 shows scanning electron microscopy images and surface areas of the hADSCs adhered to gradient PE surfaces on days 1 and 5. Most of the cells attached to L-0.5 and L-4.5 PE surfaces on day 1 had smaller surface contact areas, resulting in more rounded cell morphology. On day 5, the contact area of hADSCs was still small at L-0.5. However, hADSCs attached at L-2.5 and L-4.5 had higher surface areas, resulting in a more flattened morphology.

### 2.3. IGb mRNA

**Figure 6.**
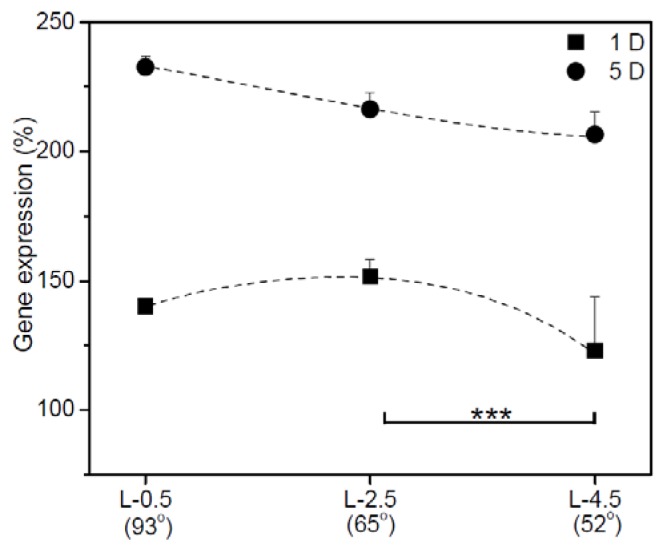
Relative expression of integrin beta 1 in hADSCs after adhesion on each gradient surface (L-0.5, L-2.5, and L-4.5) on days 1 and 5. Levels of messenger RNA (mRNA) were measured with reverse transcription-polymerase chain reaction (RT-PCR) and normalized with β-actin mRNA (^***^ *p* < 0.05).

The IGb expression of hADSCs adhering to gradient surfaces was further examined. Figure 6 shows the relative expression of IGb mRNA in hADSCs adhering to gradient PE surfaces after 1 and 5 D of culture. On day 1, the expression of IGb on the surface at L-2.5 was slightly higher than those at L-0.5 and L-4.5. On day 5, IGb gene expression on the surface at L-2.5 and L-4.5 was increased. Meanwhile, IGb expression was dramatically increased in hADSCs on the surface at L-0.5.

### 2.4. HSP70 mRNA

**Figure 7.**
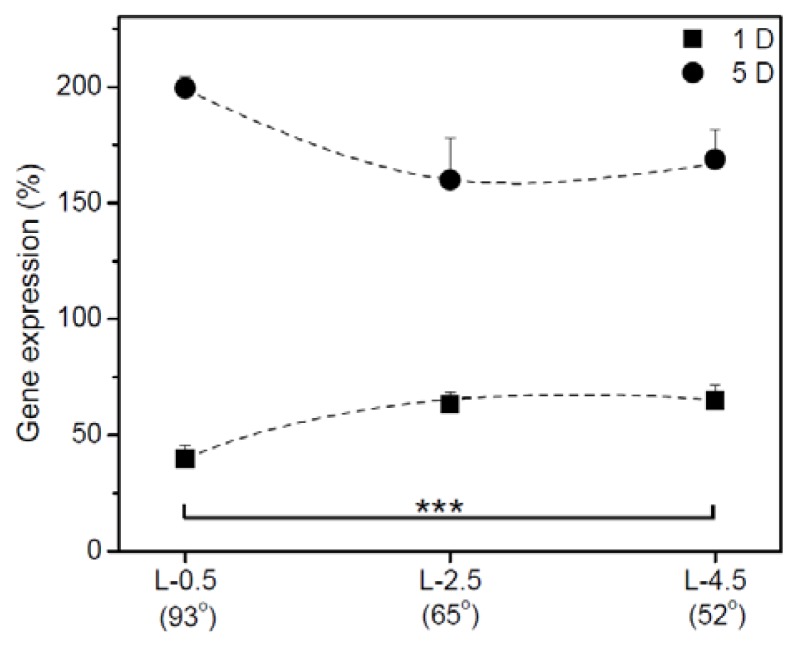
Relative expression of heat shock protein 70 in hADSCs after adhesion on each gradient surface (L-0.5, L-2.5, and L-4.5) on days 1 5. Levels of mRNA were measured with RT-PCR and normalized with β-actin mRNA (^***^ *p* < 0.05).

Figure 7 shows the relative expression of HSP70 mRNA in hADSCs adhering to gradient surfaces after 1 and 5 D of culture. The expression of HSP70 mRNA at L-2.5 and L-4.5 on day 1 was higher than that at L-0.5. The gene expression at L-2.5 and L-4.5 was increased on all surfaces on day 5. HSP70 expression was dramatically increased in hADSCs on the surface at L-0.5.

## 3. Discussion

This study investigated the cellular behavior of ADSCs on gradient surfaces. Corona treatment is a process used to prepare such surfaces [17–21]. The PE surfaces in this work were gradually oxidized during exposure to the corona. In addition, gradient oxygen-based polar functional groups can induce gradient surface charge [11,21]. hADSCs showed higher attachment on hydrophilic and rough PE surfaces. The hADSCs attached on hydrophilic surfaces exhibited extensively flattened morphology, indicating relatively strong attachment and spreading, whereas the majority of hADSCs on hydrophobic surfaces were round, indicating poor attachment. The cell proliferation rate of hADSCs on the hydrophilic and rough PE surface was higher than that on the hydrophobic surface. This adherence and proliferation as well as the attachment morphology of hADSCs were similar to those for various cell types (endothelial, Chinese hamster ovary, PC12, fibroblasts, and human BMSCs) reported in previous studies [10–12,14,30–32]. These findings indicate that the adhesion and proliferation of hADSCs may also be affected by the wettability of the surface and surface topography as well as surface charge properties.

In this study, we examined the internal gene expression of hADSCs on gradient surfaces. Integrins are transmembrane receptors that mediate the attachment between a cell and its surroundings, such as other cells or the extracellular matrix [26,27]. HSP70 is a known anti-apoptotic protein triggered in response to environmental stress such as hypoxia, oxidization, high temperature, starvation, and toxicity [28,29]. Thus, HSP70 can be an indicator of stress and apoptosis in intracellular signaling.

Therefore, we examined IGb and HSP70 expression to demonstrate the behavior of hADSCs in response to the wettability and roughness of gradient surfaces, although the results have not actually mirrored the relationship between relative expression of genes and surface properties. On the hydrophilic and rough surface, IGb expression on the first day was high, especially compared with that on the hydrophobic and smooth surface. IGb expression of hADSCs increased with the length of culture, increasing dramatically on the hydrophobic and smooth surface because hADSCs tried to attach to hydrophobic and smooth surface.

On day 1, HSP70 expression on hydrophilic and rough surfaces was higher than that on hydrophobic and smooth surfaces, but no large differences occurred between the surfaces. HSP70 expression of hADSCs increased with length of culture in a pattern similar to that of IGb expression. Furthermore, HSP70 expression increased dramatically on the hydrophobic and smooth surface, implying that the hydrophobic and smooth surface induced stress in hADSCs that led to their attachment and proliferation.

## 4. Experimental Section

### 4.1. Preparation and Characterization of Gradient Polyethylene (PE) Surfaces

A low-density PE (LDPE) sheet (thickness, 280 ± 20 μm; Hanyang Chemical Co., Busan, Korea) was used to prepare various types of wettable surfaces. The LDPE film was cut into 5-cm × 7-cm pieces, washed with 70% ethanol via ultrasonic vibration for 30 min, and dried at room temperature on a clean bench. The LDPE film was treated with a radio frequency (RF) corona discharge apparatus to prepare various wettable gradient surfaces in a manner similar to that used in our previous studies [14–21]. The corona discharge apparatus consisted of 2 key parts used to generate wettability on polymeric surfaces: a knife-type electrode connected to an RF generator that makes wettability gradient surfaces via increased power (from 10 to 35 watts at 100 kHz) and a movable sample bed (5 s as 1.0 cm/s). The electrode was placed 1.5 mm from the sample surface. Using this treatment, the sample film was continuously exposed to the corona for 5 s and developed gradient wettability on its surface.

Corona-treated surfaces were characterized by measuring the water contact angle, an indicator of surface wettability, with the sessile drop method at room temperature using an optical bench-type contact angle goniometer (Model 100-0, Rame-Hart, Inc., Succasunna, NJ, USA) Drops of purified water (3 μL) were deposited onto the corona-treated LDPE surface along the sample length using a microsyringe attached to the goniometer. For surface analysis, atomic force microscopy (AFM) measurements were carried out in tapping mode with a Nanoscope IV instrument (Digital Instruments Inc., Santa Barbara, CA, USA). The cellular behavior of ADSCs was examined along the length of the PE surface at 0.5 (L-0.5), 2.5 (L-2.5), and 4.5 (L-4.5) cm after corona treatment, with water contact angles of approximately 93º (hydrophobic surface), 65º (moderate surface), and 52º (hydrophilic surface), respectively.

### 4.2. Isolation and Characterization of hADSCs

Fresh subcutaneous adipose tissue from a young woman was donated by the Catholic University of Korea, St. Mary’s Hospital. The adipose tissue was washed twice with phosphate-buffered saline (PBS). After centrifugation at 300× *g* for 5 min, the resultant adipose tissue was digested for 30 min at 37 ºC with 15 mL 0.075% collagenase type II (Sigma, St. Louis, MO, USA), after which an equal volume of Dulbecco’s modified Eagle’s medium (DMEM; Sigma, St. Louis, MO, USA) supplemented with fetal bovine serum (FBS; Gibco, Paisley, UK) was added. The tissue debris was separated using 100-μm nylon filters. The filtered cells were suspended in DMEM with 10% FBS and then plated in 25-cm^2^ tissue culture flasks. Nonadherent cells were removed by replacing the medium after 72 h. The culture medium (DMEM supplemented with 10% FBS, 100 U/mL penicillin, and 100 μg/mL streptomycin) was changed every 3–4 days during culture. Adherent cells, hADSCs, were washed with PBS and detached with 0.05% trypsin-ethylenediaminetetraacetic acid. The hADSCs were cultured until passage 5 in this study. In the characterization, hADSCs (10^6^ cells) were washed with PBS and incubated with fluorescent conjugated antibodies (1 μg/10^6^ cells) for CD29, CD44, CD34 and CD45 (BD PharMingen, San Diego, CA, USA) in PBS (supplemented with 1% bovine serum albumin (BSA) in pH 7.5 PBS) for 30 m on ice, then washed with PBS and fixed in 1% paraformaldehyde (R & D Systems, Minneapolis, MN, USA). Cells were analyzed using a FACScan (Becton Dickinson, Franklin Lakes, NJ, USA) flow cytometer system. Cell analysis was performed using at least 10,000 events per sample. Data acquisition and analysis were then performed using Becton Dickinson Cell Quest software. The hADSCs showed positive expression of mesenchymal stem cell markers CD29 (90%) and CD44 (91.9%), negative expression of hematopoietic stem cell markers CD34 (8.4%), CD45 (0.6%), and c-kit (0.2%) and negative expression of the MHC class II protein HLA-DR (0.3%).

### 4.3. hADSCs on Gradient Surface

The hADSCs were labeled using a PKH26 Fluorescent Cell Linker Kit (Sigma) according to manufacturer instructions. Briefly, the cultured hADSCs were washed with serum-free media and centrifuged at 400× *g* for 5 min. The provided diluent C (1 mL) was added to 2 × 10^7^ hADSCs and immediately mixed with 1 mL PKH26 stock solution (4 × 10^−6^ M) in diluent C. After the mixture was incubated for 5 min at room temperature with gentle tapping, 2 mL FBS was added, and the samples were incubated for 1 min to stop the labeling reaction. Finally, the hADSCs were centrifuged at 400× *g* for 5 min, transferred to a fresh tube, and washed 3 times with complete DMEM. Labeled hADSCs (3 × 10^4^) were seeded in 24-well plates (BD Bioscience, Franklin Lakes, NJ, USA) with gradient surfaces. Immunofluorescence images were captured on days 1 and 5, and visualized under a IX81 fluorescent microscope (Olympus, Tokyo, Japan). In addition, after 1 and 2 days of culture on the PE surfaces, hADSCs were fixed for 24 h with 2.5% glutaraldehyde, dehydrated with a graded series of ethanol, coated with gold (Emscope, Model SC500K, West Sussex, UK), and analyzed by scanning electron microscopy (SEM, Hitachi Co., Model S-2250N, Tokyo, Japan). The intensity of red fluorescence expression and the area of cells attached on surface were analyzed using Image J (National Institutes of Health, Bethesda, MD, USA).

### 4.4. Cell Viability Assay

To evaluate cell viability on various wettable surfaces (L-0.5, L-2.5, and L-4.5), we cut the corona discharge films into circles, inserted them into 24-well plates, and fixed them with a silicon mold. hADSCs (2 × 10^4^ cells) were seeded onto each surface and incubated at 37 ºC in an atmosphere of 5% carbon dioxide/95% air. After 1 and 5 days, cell viability on each gradient surface was measured using a cell counting kit (CCK-8; Dojindo, Tokyo, Japan). Briefly, 50 μL CCK-8 reagent was added to the hADSCs, the plates were incubated at 37 ºC for 4 h, and then the samples were gently pipetted. An aliquot from each well (100 μL) was transferred to a 96-well plate (BD Bioscience), and absorbance at 450 nm was measured with a microplate reader (EL808 Ultra Microplate Reader; Bio-Tek Instrument, Winooski, VT, USA). All experiments were performed in triplicate, and the results were presented as means ± standard deviation.

### 4.5. RNA Extraction and Reverse Transcription-Polymerase Chain Reaction (RT-PCR)

At each time point, cells were washed with PBS, mixed with 1 mL TRIzol reagent (Invitrogen Life Technologies Co., Leek, The Netherlands), incubated for 5 min at 37 ºC, and collected in 1.5-mL tubes. The solution was mixed with 200 μL chloroform and centrifuged at 12,000× *g* for 15 min at 4 ºC. The upper aqueous phase was collected and mixed with 500 μL isopropanol, and the RNA pellet was collected via centrifugation at 12,000× *g* for 10 min at 4 ºC. The concentration and purity of RNA were determined from the ratio of the absorbance at 260/280 nm. The final concentration of total RNA was adjusted to 3 μg/μL. DNAse/RNAse-free water (Gibco BRL), oligo dT primer (Invitrogen, Carlsbad, CA, USA), deoxyribonucleotide triphosphates (Gibco), 5× first-strand buffer (Invitrogen), RNAse inhibitor (Invitrogen), and SuperScript II were sequentially added to the mixture, which was then incubated at 42 ºC for 15 min. The PCR reaction was carried out with PCR Master Mix (Elpis Biotech, Daejeon, Korea). The following primer pairs were obtained from published sequences and purchased from GenoTech (Daejeon, Korea): IGb, 5′-TATCCCATT GACCTCTACTACCTT-3′ and 5′-GGAAAGGGAATTGTATGCATCAAT-3′; HSP70, 5′-TCTGGACTGAATGTGCT TCG-3′ and 5′-ATCCCCATTTGTGGATTT CA-3′; and β-actin, 5′-ACTACCTCATGAAGATCCTC-3′ and 5′-CTAGAAGCATTTGCGGTGGACGATGG-3′. Amplification was carried out for 35 cycles (denaturation at 94 ºC for 40 s, annealing at 57 ºC for 1 min, and extension at 72 ºC for 3 min) for β-actin and IGb, and 35 cycles (denaturation at 94 ºC for 30 s, annealing at 57 ºC for 30 s, and extension at 72 ºC for 45 s) for HSP70. The PCR products were loaded onto 1.2% agarose gels, stained with ethidium bromide, and visualized with an ultraviolet transilluminator (Spectroline, Westbury, NY, USA). The fluorescence intensity of each band was measured using LabWorks™ Image Acquisition and Analysis Software (UVP, San Jose, CA, USA), and the expression of TG, IGb, and HSP70 messenger RNA (mRNA) were determined relative to that of β-actin expression.

### 4.6. Statistical Analysis

Statistical analyses were carried out with Prism 3.0 software (GraphPad Software Inc., San Diego, CA, USA). Cell proliferation and gene expression were compared using 1-way analysis of variance with Bonferroni’s multiple comparison.

## 5. Conclusions

This study examined the cellular behavior of hADSCs and wettability and roughness of gradient PE surfaces generated with corona discharge treatment. We found that the adherence of hADSCs was better on hydrophilic and rough surfaces. Adhering morphology of hADSCs on hydrophilic and rough surfaces was well stretched and flattened. hADSCs also exhibited higher proliferation on hydrophilic and rough surfaces compared to that on hydrophobic surfaces. Furthermore, we found that the transient expression of IGb and HSP70 in hADSCs on gradient surfaces depends mainly on surface wettability and roughness characteristics. Although this work showed slight difference in the gene expression behavior between 1 and 5 days, our results only reflect the relationship between transient expression of genes and the response of hADSCs to wettable materials. Thus, we believe that the results described in this study will be useful for the prediction of surface properties, wettability and roughness for implantable scaffolds.
